# Ultra-Broadband THz Antireflective Coating with Polymer Composites

**DOI:** 10.3390/polym9110574

**Published:** 2017-11-03

**Authors:** Bin Cai, Haitao Chen, Gongjie Xu, Hongwei Zhao, Okihiro Sugihara

**Affiliations:** 1 Engineering Research Center of Optical Instrument and System, Ministry of Education, Shanghai Key Laboratory of Modern Optical Systems, University of Shanghai for Science and Technology, No. 516 Jun Gong Road, Shanghai 200093, China; 167710529@st.usst.edu.cn (H.C.); gjxu@usst.edu.cn (G.X.); 2 Shanghai Institute of Applied Physics, Chinese Academy of Sciences, Shanghai 201800, China; zhaohongwei@sinap.ac.cn; 3 Graduate School of Engineering, Utsunomiya University, Utsunomiya 321-8585, Japan; oki-sugihara@cc.utsunomiya-u.ac.jp

**Keywords:** COP–TiO_2_ composite, epoxy–TiO_2_ composite, antireflection, THz, ultra-broadband, graded refractive index

## Abstract

Achieving an ultra-broadband range is an essential development direction in terahertz techniques; however, a method to cover the full terahertz band by using a highly efficient antireflection (AR) coating that could greatly increase the efficiency of terahertz radiation is still lacking. It is known that structures possessing a graded-index profile can offer a broadband AR effect, and such structures have been widely used, especially in the visible range. In this paper, first, we tuned the refractive index of a cyclo-olefin polymer (COP) by using a TiO_2_ dopant, and a polymer–TiO_2_ composite with a refractive index of 3.1 was achieved. We then fabricated a surface-relief structure with a graded-index profile by using a hot-embossing method. The structure on the silicon substrate can provide an excellent AR effect, but the working band is still limited by its scale of sag and swell. To obtain an ultra-broadband AR effect, we then proposed a flat six-layer structure; a graded-index profile was obtained by casting epoxy–TiO_2_ composites in the order of a high index to lower indices. With a very well controlled refractive index and thickness of each layer, we achieved an AR effect of <2% in the ultra-broadband of 0.2–20 THz.

## 1. Introduction

The unique properties of terahertz (THz) radiation give it great application potential in multiple disciplines, such as molecular biological science, medical imaging, astronomy, and future communication systems [1,2,3,4], and considerable research efforts have been made worldwide in the past few decades [5,6,7]. To fully exploit the utilization of the THz band, ultra-broadband THz sources [8,9] and detectors [10] are intensively studied. Compared with the significant research focusing on these active THz components, the development of antireflection (AR) coatings, especially very broadband AR coatings, still lags behind. Conventional AR coatings consist of mono- or multilayer systems whose function is based on destructive interference. The AR coating is usually a quarter-wave-thick film, which has a refractive index of $n=\sqrt{n_{substrate}}$. This kind of homogeneous AR coating can achieve low reflection, but it is restricted due to its inadaptability for broadband AR applications. With metamaterials, one can independently tailor both electric permittivity ε and magnetic permeability μ; therefore, they can be utilized to overcome the impedance ($Z=\sqrt{\varepsilon /\mu }$) mismatch at the incident interface. Although some successful AR examples using metamaterials have been proposed [11], to realize a broadband AR effect, so far the general concept of metamaterials still suffers from high loss and complexity of process and design. Another approach to achieve a very broadband AR effect is to mimic the AR structure of a moth eye, which possesses a subwavelength sag and swell structure with a gradually varying refractive index profile. To realize this kind of graded-index profile in the THz frequency, many surface structures consisting of nanotips [12], micropyramids [13], and micro-semisphere [14] arrays were proposed; these yielded minimum reflectance values of 25%, 3%, and 1.8%, respectively, with bandwidths of 1–2 THz. In this paper, we reviewed our previous work on AR structure fabrication in the THz region using refractive-index-controllable polymer composites. Following the moth eye concept, we obtained graded-index profiles by fabricating a surface-relief structure or a multilayered structure, respectively. The multilayered structure exhibits an excellent AR effect, achieving a reflectance of <2% with an ultra-broadband range of 0.2–20 THz. 

## 2. Surface-Relief Graded-Index AR Structure 

### 2.1. Polymer Composite Preparation

**Figure 1:**
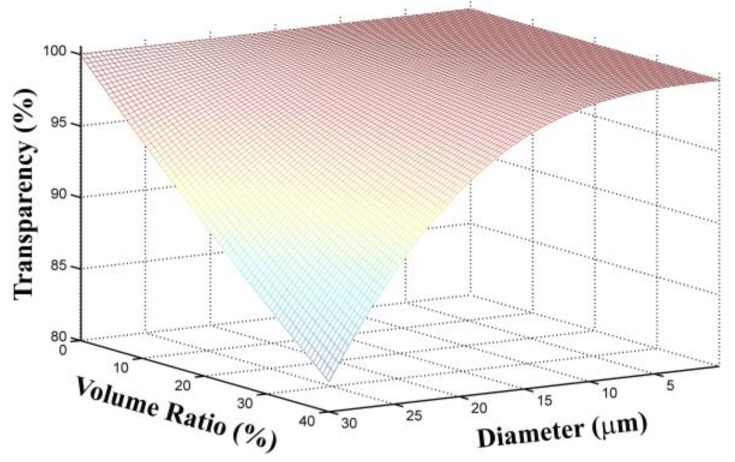
Transparency of cyclo-olefin polymer (COP)–TiO_2_ composites as a function of TiO_2_ particle diameter and the volume ratio calculated by using Rayleigh theory, where the refractive index of TiO_2_ is 6.30, that of COP is 1.52, thickness = 100 μm, and frequency = 1 THz, respectively.

We chose cyclo-olefin polymer (COP 480R, ZEONEX, Tokyo, Japan) for polymer composite fabrication because of its good solubility and broadband transparency in the THz region [15]. However, owing to its low refractive index of 1.52, there is a large Fresnel reflection loss when directly coating it onto a high refractive index substrate (e.g., Si wafer, which has a refractive index of 3.4 in the THz region). To obtain a high refractive index, we tuned the COP by adding TiO_2_ nanoparticles with a rutile crystal structure, because it has relatively low optical loss and very high permittivity in the THz region [16]. Doping particles having a large refractive index difference from that of the matrix can induce large scattering optical loss; therefore, it is necessary to investigate the relationships between transparency and doping amount or particle size. According to Rayleigh scattering theory [17,18], we calculated the transparency of COP–TiO_2_ composites as functions of TiO_2_ particle diameter and volume ratio by neglecting the absorption loss of the COP and TiO_2_; the result is shown in Figure 1. Other than in the visible region, owing to the long-wavelength nature of THz radiation, the optical performance of the composites in this regime is much less affected by scattering from particle aggregation. From the figure, we can see that, with a particle size of 20 μm and 40 vol %, the composite can still achieve >90% transparency. According to the simulation results, TiO_2_ particles with a diameter of 1 µm were used for the preparation of TiO_2_–COP composites.

**Figure 2:**
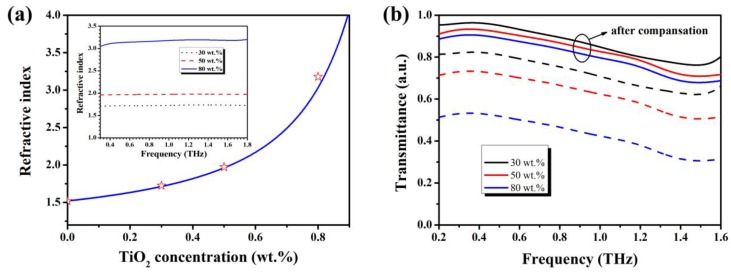
Optical properties of COP–TiO_2_ composites. (**a**) Refractive indices of the composites as a function of the doping weight ratio; the inset shows the refractive index change as a function of frequency. (**b**) Transmittance spectra of the composites.

To prepare the COP–TiO_2_ composites, first, we made a TiO_2_ microparticle toluene suspension by using a bead-milling method [17]. Then, the COPs with different masses were dissolved in the TiO_2_ suspension and mixed homogeneously by stirring for ~5–8 h. The homogenized COP–TiO_2_ suspensions were casted onto a glass substrate and baked in a vacuum oven at 60–80 °C for ~6–8 h. The fully dried COP–TiO_2_ composites can be easily peeled off from the glass substrate and are good for characterization. We investigated the transmission spectra and refractive indices of the composites via a THz time domain spectroscopy (THz-TDS) system (FiCO, Zomega Corp. East Greenbush, NY, USA). As shown in Figure 2a, we achieved refractive indices of 1.73, 1.98, and 3.10 for TiO_2_ weight ratios of 30, 50, and 80 wt %, respectively. The solid line is a fitting line obtained by using by the Bruggeman effective medium approximation [19]. Furthermore, the refractive indices of the composites remained practically constant throughout the 0.3–1.6 THz range (inset of Figure 2a); this property is beneficial for broadband THz device fabrication. From the experiments, we observed that the transmittances of the composites decrease when the particle concentrations increase, as shown in Figure 2b by the dashed lines. The high refractive indices will induce large Fresnel reflection on both sides of COP–TiO_2_ composites films. The transmittances after compensation for the reflection are also presented in Figure 2b as solid lines; we can see that the film exhibits considerable transparency in the THz region. The slightly decreased transparency could be the result of absorption by TiO_2_ particles or other impurities. 

### 2.2. Surface-Relief AR Structure Fabircation and Its AR Effect

**Figure 3:**
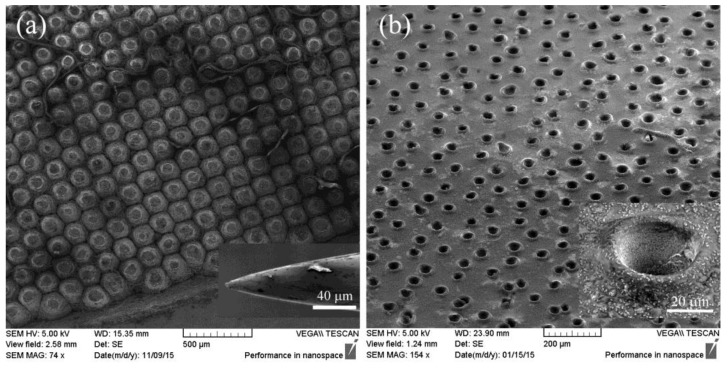
SEM images of (**a**) the top view of the homemade metallic mold (with the inset showing the needle tip used in the mold) and (**b**) the deformed COP–TiO_2_ layer obtained by using a hot embossing process (with the inset showing an enlarged image of the hole with an inverted cone shape).

The prepared COP–TiO_2_ composites not only have high refractive indices but also possess thermoplasticity as good as that of polymers. By utilizing this property, a surface-relief AR structure was fabricated by a hot-embossing technique. First, a 120-μm-thick COP–TiO_2_ composite (80 wt % TiO_2_) film was cast on a high-resistance silicon (HR-Si; resistivity >10,000 Ω·cm) wafer. The surface-relief graded-index structure was made simply by pressing a homemade metallic mold [20] into the composite at 180 °C. Scanning electron microscopy (SEM) images of the model and deformed composite layer are shown in Figure 3. We see that the composite layer was transformed into an array of holes with hole diameters in the range of 35–45 μm (Figure 3b, inset). The holes have an inverted cone shape and are surrounded with annular bulges owing to the squeezing of the mold; this results in a graded-index profile on the silicon substrate. 

**Figure 4:**
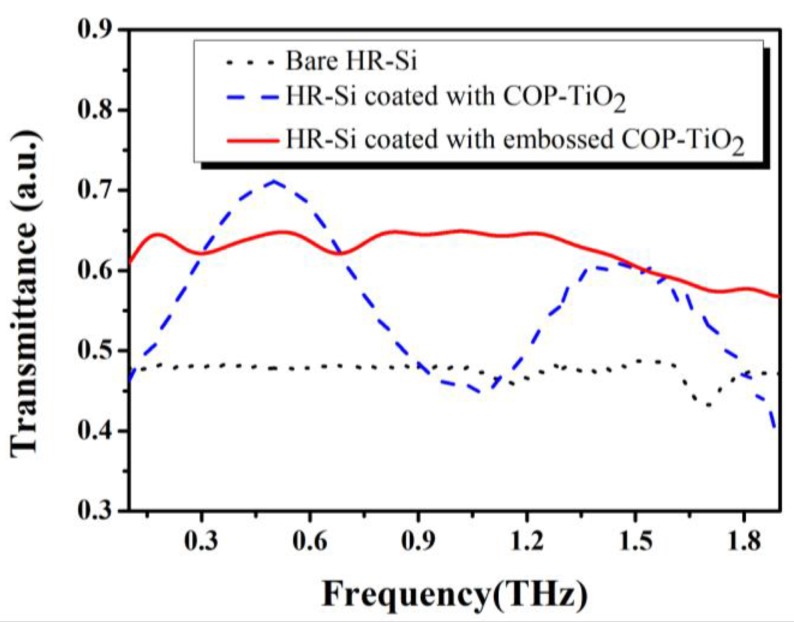
Transmission spectra of the bare high-resistance silicon (HR-Si) substrate (black dotted line), an HR-Si substrate coated with a flat COP–TiO_2_ layer (blue dashed line), and an embossed COP–TiO_2_ layer (red solid line).

The AR effects of the samples were investigated by using THz-TDS, and the results are shown in Figure 4 (where the reflection peaks in their time-domain spectra, which originate from the Fabry–Perot effect, were cut off to smoothen the experimental results in the frequency domain). The black dotted line is the transmission of an HR-Si substrate without any coating. The blue dashed line represents the transmission of a single flat COP–TiO_2_ composite layer (120 μm, *n* = 1.73) coated HR-Si substrate, which has two enhanced peaks that appear at 0.50 THz (71%) and 1.45 THz (61%), respectively. This result is consistent with that of classic destructive interference theory. The COP–TiO_2_ composite layer sample (solid red line) exhibits a broadband AR effect throughout the whole measurement range, achieving an average transmittance of up to 60% throughout the frequency range from 0.2 to 1.4 THz [21]. Here, it must be noted that the sample suffered from an additional Fresnel loss of ~28% during the measurement because of rear-side (noncoating) reflection.

## 3. Surface Flat Graded-Index AR Structure

### 3.1. Structure Design and Fabrication

**Figure 5:**
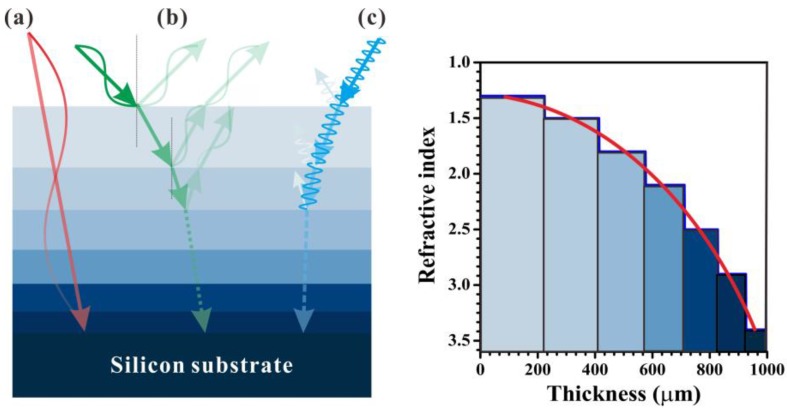
(**Left**) Schematic diagram of the ultra-broadband antireflection (AR) coating. The bottom substrate is a Si wafer, and the refractive indices were changed gradually with a flat six-layer structure. The red (a), green (b), and blue (c) lines stand for the propagated behaviors of different wavelengths in the terahertz range. (**Right**) Refractive index profile of the six-layer structure as a function of the thickness of each layer.

The graded-index profile achieved by a surface-relief structure can provide a broadband AR effect; however, the effect is still limited by the size of the sag and swell; the AR effect will be suppressed when incident wavelengths are much larger or smaller than the structure size. For THz radiation, the wavelength difference could be as large as 100-fold, e.g., 0.1 and 10 THz, thus there still remains a huge hurdle in realizing a full THz band AR. To achieve a structure that would correspond to the full THz bandwidth, we proposed a flat six-layer structure [22] for ultra-broadband AR, as shown in Figure 5 (left). To mimic the graded-index profile, the refractive indices of the six layers are varied from 1.3 to 2.9 individually. Unlike the surface-relief AR structures mentioned above, there are no sag and swell structures in the plane; therefore, the AR effect can be wavelength independent over a very broad range. 

The refractive index of each layer is determined using

(1) $$\frac{n_{air}}{n_{1}}\approx \frac{n_{1}}{n_{2}}\approx \frac{n_{2}}{n_{3}}\approx \frac{n_{3}}{n_{4}}\approx \frac{n_{4}}{n{\text{​}}_{5}}\approx \frac{n_{5}}{n_{6}}\approx \frac{n_{6}}{n_{Si}}$$

where *n*_1_–*n*_6_ are the refractive indices of the first to sixth layers and *n_air_* and *n_Si_* are the refractive indices of air and the silicon substrate, respectively. The refractive index as a function of the thickness of each layer is also shown in Figure 5 (right). The thickness *T_i_* of each layer is determined by destructive interference theory in which the frequency was set to 5 THz. To cover the low terahertz band, a comparably thick layer is identified by *T_i_* = 19λ/4*n_i_*, where λ is 60 μm (5 THz) and *n_i_* is the refractive index of each layer.

The flat six-layer structure contains three different mechanisms to achieve an ultra-broadband AR effect. In the case of low frequencies, the thickness of each layer is smaller than the incident wavelength, the multilayers act like a graded-index media, and the reflective interface is nonsusceptible, which results in low reflection, as shown by the red lines (a) in Figure 5. In the case of high frequencies, the thickness is much larger than the wavelength, and the THz wave follows the Fresnel equation and is reflected on each interface. However, the refractive index difference for each adjacent layer is so low that most of the THz waves can pass through the layers with very low reflection, as shown by the blue lines (c). An incident wavelength λ = 4*n_i_T_i_*/*m* (where *m* is an odd number) that satisfies destructive interference AR conditions also results in a very good AR effect, as shown by the green lines (b).

### 3.2. Materials and Device Fabircation

For the preparation of the composites, instead of COP, we used epoxy resin (3,4-epoxy-cyclohexylmethyl 3,4-epoxycyclohexane carboxylate, ECC) with hardener *m*-xylylenediamine as the polymer matrix owing to its better suitability for the multilayer coating process. The ECC resin has a glass transition temperature of 135 °C and a decomposition temperature of 370 °C, which are high enough for the multilayer preparation process mentioned below. The ECC resin has a refractive index of 1.5 at 1 THz, and we tuned the refractive index of the resin by varying dopants and doping concentrations. For high-refractive-index composites, we used TiO_2_ nanoparticles to achieve the targeting refractive indices. First, the TiO_2_ nanoparticles are bead-milled with a surface modifier (Disperbyk-180, BYK additives & Instruments, Wesel, Germany) for dispersion in an ethanol solvent to prevent the aggregation of particles. Then, an equivalent proportion of ECC and hardener are dissolved and stirred for 2 h in the TiO_2_ suspension. Finally, the composite material is cast on a substrate and cured at 100 °C. According to the Bruggeman effective medium approximation, we achieved refractive indices of 1.8, 2.1, 2.5, and 2.9, by controlling the weight ratios of TiO_2_ to 15%, 32%, 46%, and 60%, respectively. To achieve the low refractive index of 1.3, we used hollow polystyrene (PS) microspheres (Expancel DE, AkazoNobel, Amsterdam, the Netherlands) as the dopant of the polymer. The PS microspheres have a diameter range from 20–40 μm, and the thickness of the sphere shell is ~0.1 μm; thus, their addition can greatly decrease the refractive index of the composite. The PS microspheres were first wetted with ethanol, mixed with ECC and hardener in a weight ratio of 1:1, and stirred for 2 h for homogenization. After curing at 60 °C, this PS microsphere–epoxy layer has a refractive index of 1.3. Here, we have to mention that the refractive indices are all at the band near 1 THz. It is impossible to keep the refractive indices constant throughout the full THz band; however, the trend exhibited by the gradient of the refractive index does not change.

**Figure 6:**
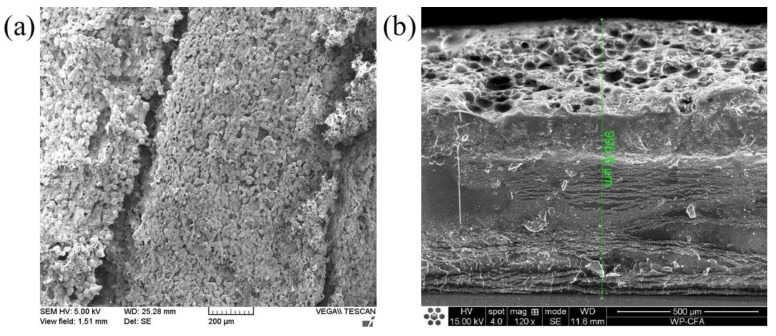
SEM images of the flat six-layer AR structure: (**a**) top view; (**b**) cross section.

The composites were cast and cured on a silicon substrate in the order of a high refractive index to a low refractive index. The casting volume and the casting surface area were very well calculated to control the thickness of each layer precisely. The theoretical thicknesses as well as real thicknesses are summarized in Table 1. The average thickness deviation is <5%. The layers at the top and bottom have comparably large deviation values owing to the large amount of the dopants that increased the adhesiveness and resulted in difficulties in controlling the thickness. SEM images from a top view and a cross-section of the device are shown in Figure 6. The hollow PS microspheres can be easily identified; however, the other layers are not as clearly distinguished because of their similar states. The device has quite a coarse surface, with an average roughness of dozens of micrometers, which will benefit its AR effect. 

### 3.3. AR Effect of the Flat Six-Layer Structure

**Figure 7:**
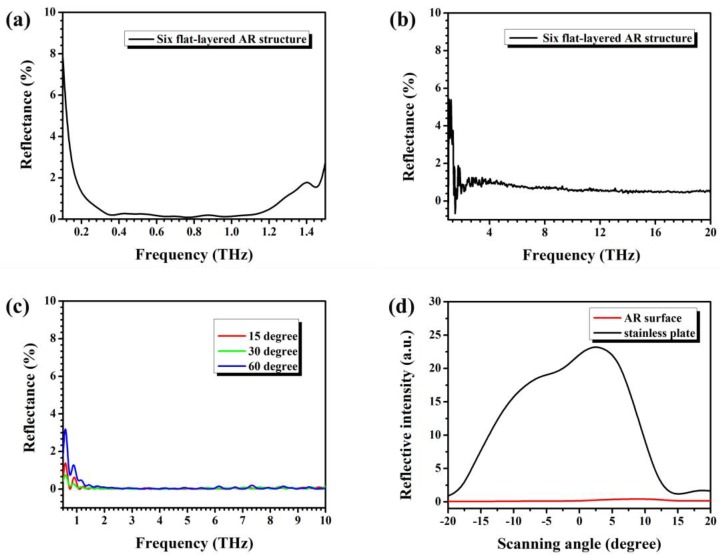
AR effects of a flat six-layer structure. (**a**) Reflection spectrum from 0.1 to 1.5 THz measured by using THz–TDS. (**b**) Reflection spectrum from 1.5 to 20 THz measured by using FT-IR. (**c**) Reflection spectra from 0.5 to 10 THz with different incident angles simulated by CST Microwave Studio. (**d**) Surface scattering intensities as a function of detection angle.

The reflective spectra of the flat six-layer structure were investigated by using THz-TDS and a Fourier-transform infrared (FT-IR) spectroscope (Bruker V80, Billerica, MA, USA), respectively, where the THz-TDS was for the range 0.1–1.5 THz and the FT-IR spectroscopy was for the range 1.5–20 THz. The measured results are presented in Figure 7a,b. By combining Figure 7a,b, we can see that the multilayered AR structure can realize an AR effect of <2% over the range of 0.2–20 THz. We also noticed that the reflectance at 0.1–0.2 THz increased quickly, because the incident wavelengths became far larger than the thickness of the structure. To investigate the incident angle dependency of the AR effect, we simulated the AR effect of the device using CST Microwave Studio. In the simulation, the incident angles were varied from 15° to 60°, and the calculation range was set to 0.1–10 THz; the results are shown in Figure 7c. From the figure, we can see that, when the frequency >1 THz, the device maintains a very low reflective rate and exhibits insensitivity to the incident angle from 0° to 60°. In the case of frequency <1 THz, although the reflectance is still maintained at quite a low level, the reflection increases rapidly and exhibits some degree of oscillation. The increase in the reflectance is mainly due to the incident angle increasing according to the Fresnel formula. 

To evaluate the scattering features, a terahertz quantum cascade laser (QCL) with a 4.2-THz center frequency and a 200-GHz bandwidth was used to irradiate the multilayered AR structure, and the scattered terahertz laser light intensity was measured by a Golay cell. The output voltage of the Golay cell was recorded as the laser intensity, and the direct output of the QCL was 139 mV. To investigate the scattering property of the multilayer, we swung the Golay cell ±20° from the reflective angle. As a reference, a coarse stainless-steel plate was measured for comparison; the results are shown in Figure 7d. From Figure 7d, we can see that, for the stainless plate, the dependence of the scattering intensity on the deviated angle roughly follows a Gaussian dispersion (black curve), and the maximum scattered value is 23.5 mV, which means ~16.90% of the incident THz radiation was scattered at the reflective angle. The red line in Figure 7d is the scattering signal of our ultra-broadband AR structure. Compared with the stainless-steel plate, our device exhibits very low scattering intensity and very weak angle dependency. The maximum scattered amplitude is only 0.47 mV. Note that the background noise level is ~0.1–0.2 mV; thus, only 0.19–0.27% can be the maximum scattering; this result is consistent with previous FT-IR spectroscopy results.

## 4. Conclusions

COP–TiO_2_ and epoxy–TiO_2_ composites were used to fabricate an AR coating in the THz range in this work. The refractive indices of the composites can be easily controlled by varying the amount and concentration of TiO_2_ dopants, which is important for matching refractive indices of different materials. By utilizing the thermal deformation property of the COP–TiO_2_ composite, we successfully fabricated an antireflection structure via a hot-embossing process. The structure provides a far broader bandwidth for the AR effect (0.2–1.6 THz, 7% reflection) than that of the traditional homogeneous AR layer. A flat six-layer structure with a graded-index profile was designed to overcome the shortcomings of the surface-relief structure. This multilayered structure exhibits an excellent AR effect of <2% with an ultra-broadband range of 0.2–20 THz. Furthermore, the AR effect of the structure is not limited by the incident angle, and the structure maintains a good AR effect from 0° to 60° without an obvious decrease. Devices based on polymer composites have many advantages, including ease of processing, low cost, and flexibility, and therefore they have large potential application in thermal detectors, microbolometers, and imaging systems to terahertz stealth fields.

## Figures and Tables

**Table 1:** Refractive index and thickness of each layer in the flat six-layer AR structure.

Layer	1st	2nd	3rd	4th	5th	6th
Refractive index	1.3	1.5	1.8	2.1	2.5	2.9
Theoretical thickness (μm)	224	190	161	136	116	98
Real thickness (μm)	238	194	158	134	116	114
Thickness deviation	6.3%	2.1%	1.9%	1.5%	0	16%
